# Passivation Mechanism in Highly Luminescent Nanocomposite‐Based CH_3_NH_3_PbBr_3_ Perovskite Nanocrystals

**DOI:** 10.1002/smsc.202400529

**Published:** 2025-01-22

**Authors:** Jaume Noguera‐Gómez, Víctor Sagra‐Rodríguez, Vladimir S. Chirvony, Miriam Minguez‐Avellan, Mahesh Eledath‐Changarath, Juan F. Sánchez‐Royo, Juan P. Martínez‐Pastor, Pablo P. Boix, Rafael Abargues

**Affiliations:** ^1^ Instituto de Ciencia de los Materiales de la Universidad de Valencia (ICMUV) 46980 Paterna València Spain; ^2^ Instituto de Tecnología Química Universitat Politècnica València‐Consejo Superior de Investigaciones Científicas Av. dels Tarongers 46022 València Spain

**Keywords:** emission, nanocomposite, nanocrystals, perovskite

## Abstract

Water exposure significantly impacts the structure and photoluminescence (PL) of metal halide perovskites. However, humid conditions can enable the in situ synthesis of methylammonium lead bromide (MAPbBr_3_) perovskite nanocrystals (NCs) within a nickel acetate matrix, achieving PL quantum yields (PLQY) of up to 80%. The water‐driven formation and transformation of MAPbBr_3_ is presented, highlighting the crucial role of acetate. Comprehensive optical and structural analyses reveal that low relative humidity (RH < 20%) favors the formation of non‐emissive MA_4_PbBr_6_ (0D) and hydroxide species (PbBrOH, OH^−^) . Exposure to higher RH induces a structural reorganization from 0D MA_4_PbBr_6_ to 3D MAPbBr_3_ via a MABr‐stripping mechanism, forming NCs with enhanced PLQY. Removing ambient humidity quenches PL, a process that is reversible due to hydroxide‐mediated reactions controlled by dual acid‐base nature of the acetic acid/acetate system. Unlike previous reports, the findings reveal that hydroxide ions reversibly bind to NCs, passivating traps and improving stability. Acetate's basicity plays a critical role in generating OH^−^, promoting the passivation, stability, and enhanced optical properties of the perovskite nanocomposites.

## Graphical abstract

A comprehensive investigation into the humidity‐driven crystallization dynamics of methylammonium lead bromide (MAPbBr_3_) NCs is presented, showing that increased RH promotes the transformation of 0D MA_4_PbBr_6_·2H_2_O into 3D MAPbBr_3_. The results emphasize the pivotal role of acetate basicity and in situ‐generated OH^−^ in passivating NCs, enhancing their stability, and optimizing optical properties.

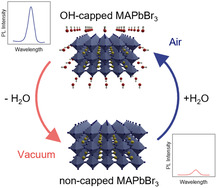

## 1 Introduction

Over the past decade, colloidal nanocrystals (NCs) of 3D metal halide perovskites, with the chemical formula APbX_3_ (where A = CH_3_NH_3_^+^ (MA^+^), CH(NH_2_)_2_^+^ (FA^+^ or Cs^+^ and X = Cl^−^, Br^−^, or I^−^), have garnered significant attention. Typically synthesized via hot injection methods in organic solvents, these NCs exhibit exceptional photoelectronic properties, including high photoluminescence quantum yield (PLQY), narrow emission spectra, tunable emission wavelengths, and high defect tolerance,^[^ ^1^, ^2^, ^3^, ^4^, ^5^ ^]^ All these features make them suitable for various applications, such as light‐emitting diodes^[^ ^6^, ^7^ ^]^ and solar cells.^[^ ^8^, ^9^, ^10^ ^]^ However, despite exhibiting excellent photophysical properties in solution, these colloidal NCs face several challenges when implemented in functional thin films. Specifically, they experience significant drawbacks in their photoluminescence (PL) performance and, consequently, their overall optoelectronic properties, as well as faster degradation. The reasons postulated for such functional and structural instabilities include the loss of passivating organic ligands in solid‐state films of perovskite NCs as well as their interaction with moisture. H_2_O molecules diffuse within the NC lattice and form hydrogen bonds with electronegative atoms (e.g., nitrogen and halogen). This induces a structure degradation into its precursors or their derivatives,^[^ ^11^, ^12^, ^13^, ^14^ ^]^ which impacts the thin‐film quality and device performance.^[^ ^13^, ^15^, ^16^ ^]^ Thus, developing stabilization and passivation strategies is crucial for creating robust materials suitable for future applications.^[^ ^17^, ^18^ ^]^

In contrast, several reports highlight the positive role of H_2_O molecules in the perovskite crystallization.^[^ ^19^, ^20^, ^21^, ^22^ ^]^ Some describe its role in the reorientation of the crystals,^[^ ^20^ ^]^ while others point out that ambient water can facilitate the generation of lead derivatives such as PbBrOH.^[^ ^23^ ^]^ Although the passivation mechanism in these bromide systems is not fully understood, PbBrOH is often suggested to significantly contribute to an active passivation, enhancing both stability and luminescence of 3D perovskite NCs.^[^ ^24^ ^]^ Besides, moisture plays a critical role in triggering a phase transition from initially formed low‐dimensional 0D perovskites (A_4_PbX_6_) to their 3D counterparts (APbX_3_)^[^ ^25^, ^26^ ^]^ via the halide cation (AX)‐stripping mechanism facilitated by their solubility in water.^[^ ^27^, ^28^, ^29^, ^30^, ^31^, ^32^ ^]^ Similar to the PbBrOH, the higher bandgap 0D phase can have an active role in the passivation of the 3D NCs.^[^ ^33^ ^]^

Beyond these effects, we recently reported that ligand‐free MAPbBr_3_ perovskite NCs in situ synthesized in a nickel acetate (Ni(AcO)_2_) matrix under humid atmospheres displays very intensive and stable photoluminescence (PLQY ≈ 80%).^[^ ^34^ ^]^ A key finding was the significant role of the relative humidity (RH) as a crucial factor governing the crystallization process, allowing for fine‐tuning the NC formation and optimizing their optical properties. However, the mechanism underlying such exceptional optical properties and the crystallization dynamics remained not fully understood.

Here, we demonstrate that in the aforementioned MAPbBr_3_ nanocomposites, 0D phase MA_4_PbBr_6_ NCs are initially formed at low‐humidity conditions. The interaction with ambient humidity triggers two concurrent processes that determine the properties of the film: 1) the initial 0D MA_4_PbBr_6_ transition into the 3D MAPbBr_3_ NCs; and 2) the generation of hydroxide ions (OH^−^) through an acid–base reaction with H_2_O in the acetate‐rich environment, which improve passivation and enhance the optoelectronic properties of the 3D nanocrystals. Comprehensive studies on the PL performance of the OH‐passivated NCs reveal that the OH^−^ is in equilibrium with the ambient humidity and can be reversibly unbound and bound when exposed to vacuum and humidity, respectively. As a result, the 3D MAPbBr_3_ NCs exhibit intense green PL with enhanced stability, ascribed to the strong binding of hydroxide ions to the NCs surface.

## 2 Results and Discussion

**Figure 1:**
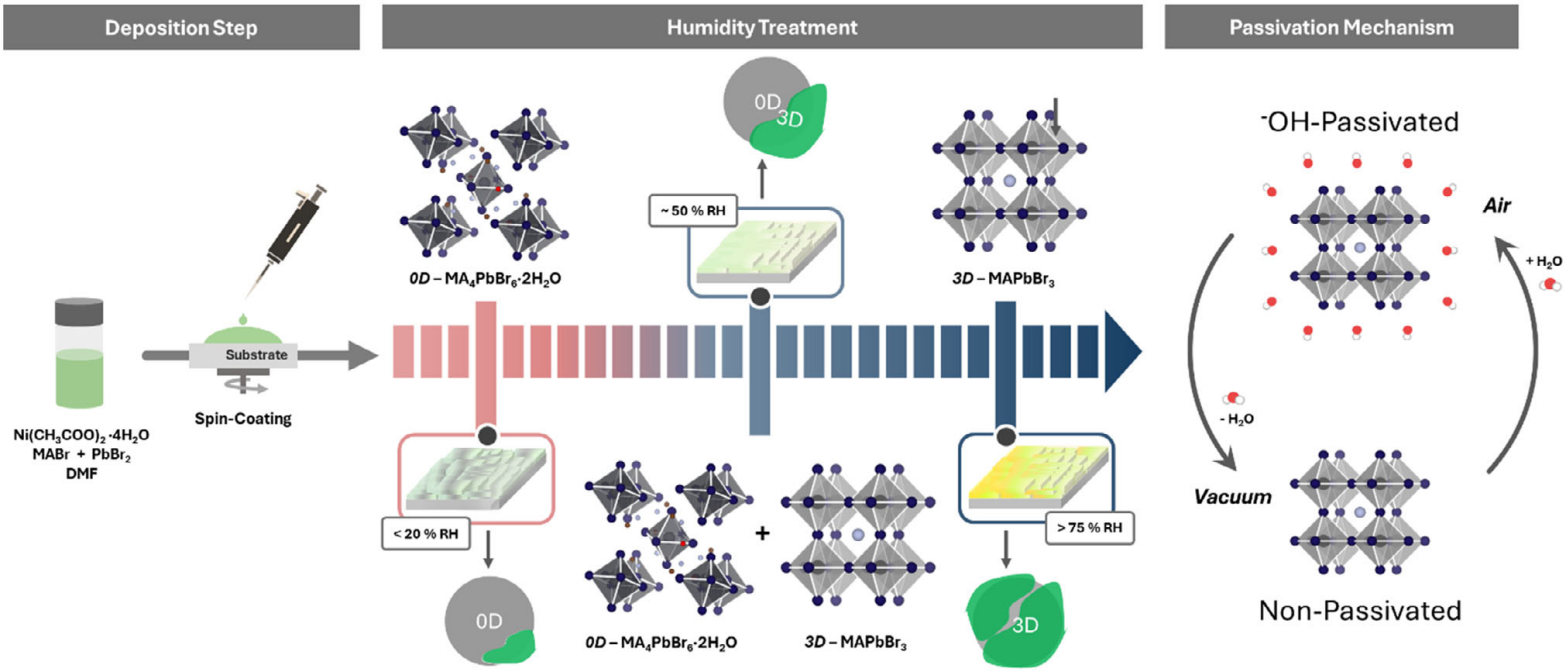
Schematic illustration of the MAPbBr_3_ nanocomposite crystallization process, highlighting the phase transformation triggered upon humidity exposure and the OH passivation mechanism governing their performance.

The formation of the nanocomposite films, based in situ synthesis of MAPbBr_3_ NCs inside a Ni(AcO)_2_ matrix, requires the presence of water to initiate the 3D perovskite crystallization, as outlined in our prior studies.^[^ ^34^, ^35^ ^]^ To gain insight into the exact role of water on the crystallization and passivation mechanism (**Figure** **1**), we focus on the influence of RH on nanocomposite on samples at a 1:0.25 molar ratio of Ni(AcO)_2_:MAPbBr_3_ (see Experimental Section for details). RH exposure is crucial not only for the growth of perovskite NCs, as it determines their size, but more importantly, for triggering structural phase transformations in the NCs, as explained below.

**Figure 2:**
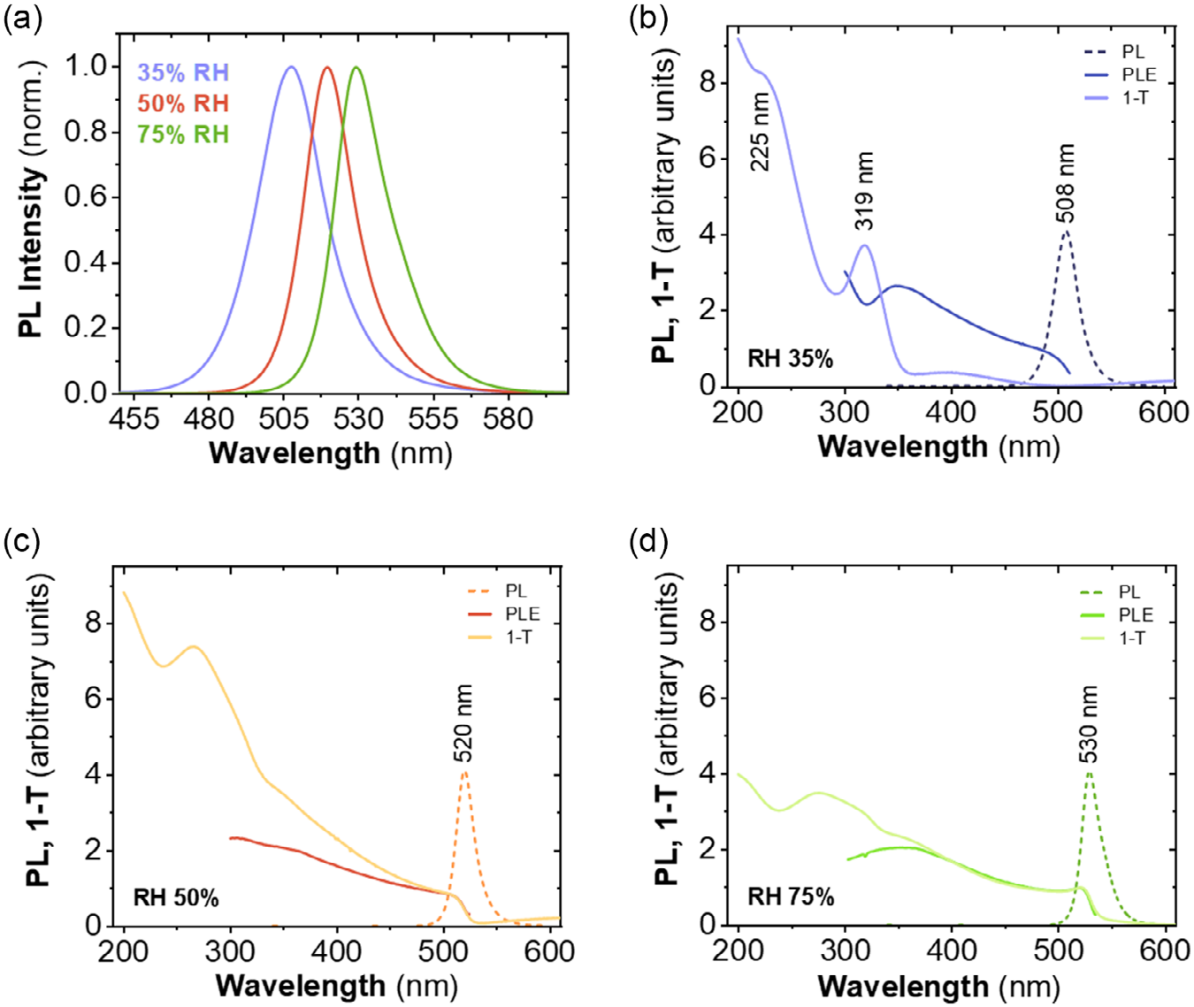
a) PL spectra of MAPbBr_3_–Ni(AcO)_2_ nanocomposites prepared under atmospheric humidity levels of 35% (sample 508), 50% (sample 520), and 75% (sample 530). b–d) PL, PLE, and 1‐T (absorptance) spectra of the aforementioned three samples.

The films deposited under low RH conditions (<20% RH) do not display PL response, yet bright green luminescence can readily be observed upon exposure to higher RH. To understand this transition, we exposed the samples to various RH (details in Experimental Section). **Figure** **2**a presents the PL spectra of three samples prepared at RH of 35, 50 and 75%, displaying a clear shift toward lower energies as humidity increases. PL, PL excitation (PLE), and 1‐Transmittance (1‐T) spectra for the same samples are represented in Figure 2b–d.

When PLE and (1‐T) signals originate from the same material, both spectra should approximately match for nanocrystals, as is the case of the RH 75% sample from 375 to 530 nm (Figure 2d). In contrast, a clear difference between PLE and (1‐T) can be observed for the sample generated at the lowest RH (35%) (Figure 2b). Note that while the PLE spectrum of the 35% RH sample is qualitatively similar to that of RH 50 and 75% RH samples, the (1‐T) spectra differ significantly from each other. (1‐T) spectrum of 35% RH sample shows intense absorption bands at 225 and 320 nm and a weak broad band at 400 nm. We can conclude that a significant part of the 35% RH sample is not the luminescent 3D perovskite MAPbBr_3_, unlike the samples formed at higher RH. Instead, 35% RH sample contains another product with a distinct absorption band at 320 nm. This absorption band is concomitant to a dip in the PLE spectrum of the MAPbBr_3_ nanocomposite (Figure 2d), indicating that the additional phase is not photoactive and it acts only as a passive light absorber that screens the excitation of the luminescent 3D perovskite. The broad absorption band at 400 nm may, at least in part, belong to the luminescent 3D perovskite.

In the case of MAPbBr_3_ nanocomposite at 50% RH (Figure 2c), the PLE and (1‐T) spectra match in the region of 475–520 nm and deviate at shorter wavelengths. The shape of (1‐T) presents the fingerprint of luminescent 3D perovskite MAPbBr_3_, yet with significant contributions from some other products (absorption maximum at 275 nm, shoulder at 360 nm).

**Figure 3:**
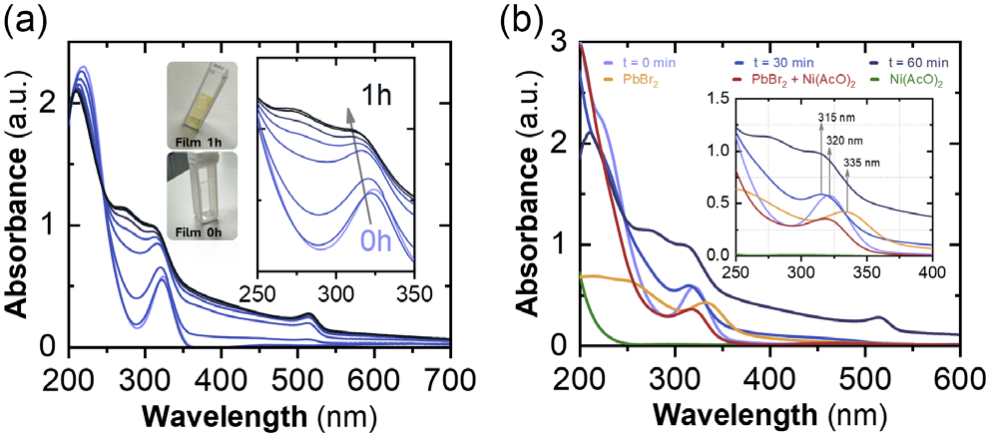
a) Absorption for a PbBr_2 _+ MABr + Ni(AcO)_2_ nanocomposite film exposed to 75% RH and 25 ºC for 1 h. b) Absorption spectra of PbBr_2_, Ni(AcO)_2_, PbBr_2 _+ Ni(AcO)_2_, and PbBr_2 _+ MABr + Ni(AcO)_2_ forming the nanocomposite (75% RH, exposure times from 0 to 60 min).

To further elucidate the RH effect on the optical properties of MAPbBr_3_–Ni(AcO)_2_ nanocomposites, we have monitored the absorbance spectra from 200 to 700 nm for different RH and exposure times. **Figure** **3**a reveals the evolution in a 75% RH environment. Only the characteristic absorption band at 320 nm is observed during the first minutes of exposure.

By increasing the exposure time a secondary absorption band seems to appear at around 275 nm, producing an effective blue shift of the 320 nm absorption band, as observed in the inset of Figure 3a, further discussed below. Simultaneously, the characteristic absorbance band of the 3D‐MAPbBr_3_ semiconductor at ≈520 nm grows, reaching its maximum intensity after 1 h of exposure.

Absorbance spectra of PbBr_2_, Ni(AcO)_2_, PbBr_2_ + Ni(AcO)_2_ films, and Ni(AcO)_2_–MAPbBr_3_ nanocomposites exposed at 75% RH for different times are shown in Figure 3b. The absorbance spectrum of a PbBr_2_ thin film (orange line) exhibits a peak at ≈335 nm, undiscernible in the other samples. Similarly, a Ni(AcO)_2_ thin film (green line) shows no absorption band within the studied spectral range. However, a PbBr_2 _+ Ni(AcO)_2_ thin film (red line) exhibits a similar absorption band than the one observed for PbBr_2_, but shifted to 320 nm. When comparing these results with the absorbance of MAPbBr_3_ nanocomposites exposed to 75% RH for different times (see Figure 3b), the 320 nm band is already visible from *t* = 0 min when no signs of 3D MAPbBr_3_ are evident. At *t* = 30 min, the absorbance spectrum still contains a band at 320 nm, yet the increase of absorption in the 250–320 nm region and the tail in the 350–500 nm region are characteristic of the 3D MAPbBr_3_. Thus, we attribute the band at 320 nm, distinguishable in the MAPbBr_3_ nanocomposites at various time intervals, to the wide bandgap of PbBrOH structure.^[^ ^36^, ^37^, ^38^, ^39^ ^]^ This effect is induced by the presence of crystalline water in Ni(AcO)_2_·4H_2_O, as supported by the results from co‐depositing PbBr_2_ and Ni(AcO)_2_ only (see Figure 7, Step (2)) that blue shifts the absorption band from 335 to 320 nm compared to the bare PbBr_2_ sample.

A more detailed analysis of spectral shifts observed in the absorption band at 315–335 nm upon longer exposures to a RH 75% (see inset of Figure 3b) suggests the existence of additional compounds in the films. Indeed, in the case of the MAPbBr_3_–Ni(AcO)_2_ nanocomposites at *t* = 0 min, the maximum of the absorption band is located at 320 nm, while in the case of *t* = 30 min is at 315 nm. We correlated this blue shift of the UV absorption band maximum to forming a 0D phase of the MA–Pb–Br family, MA_4_PbBr_6_, characterized by a clear peak at 310 nm.^[^ ^30^, ^40^, ^41^ ^]^ As suggested in several studies,^[^ ^40^, ^42^ ^]^ the transformation of 0D MA_4_PbBr_6_ to 3D MAPbBr_3_ upon interaction with water is driven by an MABr‐stripping reaction (see Figure 7, Step (3)). This mechanism highlights the role of water in destabilizing the 0D structure, facilitating the removal of MABr, and promoting the formation of the 3D perovskite phase. Thus, after the deposition at low RH (<20% RH), a nanocomposite of 0D MA_4_PbBr_6_ NCs embedded in Ni(AcO)_2_ is formed. Upon exposure to higher RH, 0D NCs are transformed into 3D MAPbBr_3_ perovskite structures, as shown in Figure 1 scheme. Unlike Cs_4_PbBr_6_, MA_4_PbBr_6_ is only stable as a complex with two water molecules, forming the structure MA_4_PbBr_6_·2H_2_O.^[^ ^30^, ^40^, ^43^ ^]^ Therefore, the waters of crystallization contained in the Ni(AcO)_2_·4H_2_O precursor appear crucial for forming MA_4_PbBr_6_·2H_2_O at early crystallization stages, even at low RH. Consequently, the observed absorbance bands around 310–330 nm strongly support the coexistence of MA_4_PbBr_6_·2H_2_O 0D perovskite and PbBrOH in the nanocomposites. Interestingly, while similar 3D/0D combinations are often defined as a type I heterostructure with funneling that enhances radiative recombination,^[^ ^33^ ^]^ no effective energy transfer can be observed from the PLE measurements. This suggests that the 0D phase and PbBrOH act as neutral UV filters.^[^ ^37^, ^44^ ^]^ It is thus crucial to gain insights into the structural and compositional details of the coexistent phases.

SEM images of MAPbBr_3_–Ni(AcO)_2_ nanocomposites show a uniform, dense structure with smooth surfaces, as seen in cross‐sectional and top‐view images (Figure S1, Supporting Information). Efforts to capture TEM images of the native unexposed structures were hindered by challenges associated with the sample preparation process. Though, upon exposure to 75% RH, TEM images reveal nanoparticles associated with 3D MAPbBr_3_ perovskites where no additional structures are observed. Further TEM experiment details are discussed in the Supporting Information.

**Figure 4:**
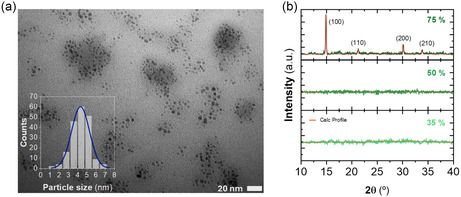
a) TEM Image and particle size distribution of MAPbBr_3_ nanocomposites prepared at 75% RH b) XRD patterns of as‐synthesized MAPbBr_3_ nanocomposites under different relative humidity conditions.

The XRD diffraction peaks of the nanocomposites exposed to 50% and 75% RH (**Figure** **4**b) correspond to the cubic P*m*‐3*m* crystal phase of MAPbBr_3_ (JCPDS no. 00‐0105). Specifically, peaks at 15°, 21.2°, 30.2°, and 33.9º align with the (100), (110), (200), and (210) crystal planes, respectively. The nanocomposite films prepared at <20% RH exhibit no crystalline patterns, confirming that MAPbBr_3_ is not formed at low RH. This is consistent with the optical properties observed in Figure 2, in which the presence of 3D NCs is associated with high RH conditions (>50%). The incorporation of a relatively low amount of perovskite within the amorphous matrix (in a 0.25:1 molar ratio MAPbBr_3_:Ni(OAc)_2_) hinders the detection of small and poorly crystalline byproducts, such as 0D perovskites and PbBrOH at early stages, using XRD. To address this limitation, we employed complementary characterization methods.

To further investigate this matter, we have employed Attenuated total reflectance Fourier transform infrared spectroscopy (ATR‐FTIR) to investigate the influence of humidity on the nanocomposite structure. Unlike XRD, which confirmed MAPbBr_3_ perovskite formation only at a RH exceeding 50%, ATR‐FTIR offered deeper insights into the compositional groups present (Figure S2, Supporting Information). The O–H vibrational band, centered at 3600–3250 cm^−1^, is detectable across all samples but displays a pronounced increase in intensity as humidity increases, ultimately surpassing the N–H band (≈3250–3000 cm^−1^). This suggests the incorporation of OH species into the layer early on. Additionally, the pronounced increase in the O–H band intensity with rising humidity provides further evidence for the adoption of an OH‐capped configuration by the 3D perovskites. This process, likely associated with an OH‐driven passivation mechanism (see Figure 7, Steps 4 and 5), is further elaborated in the section dedicated to the OH‐driven passivation mechanism.

Moreover, XPS can provide additional information to complement the optical observations related to 0D to 3D perovskite phase transformation and the OH passivation mechanism.

**Figure 5:**
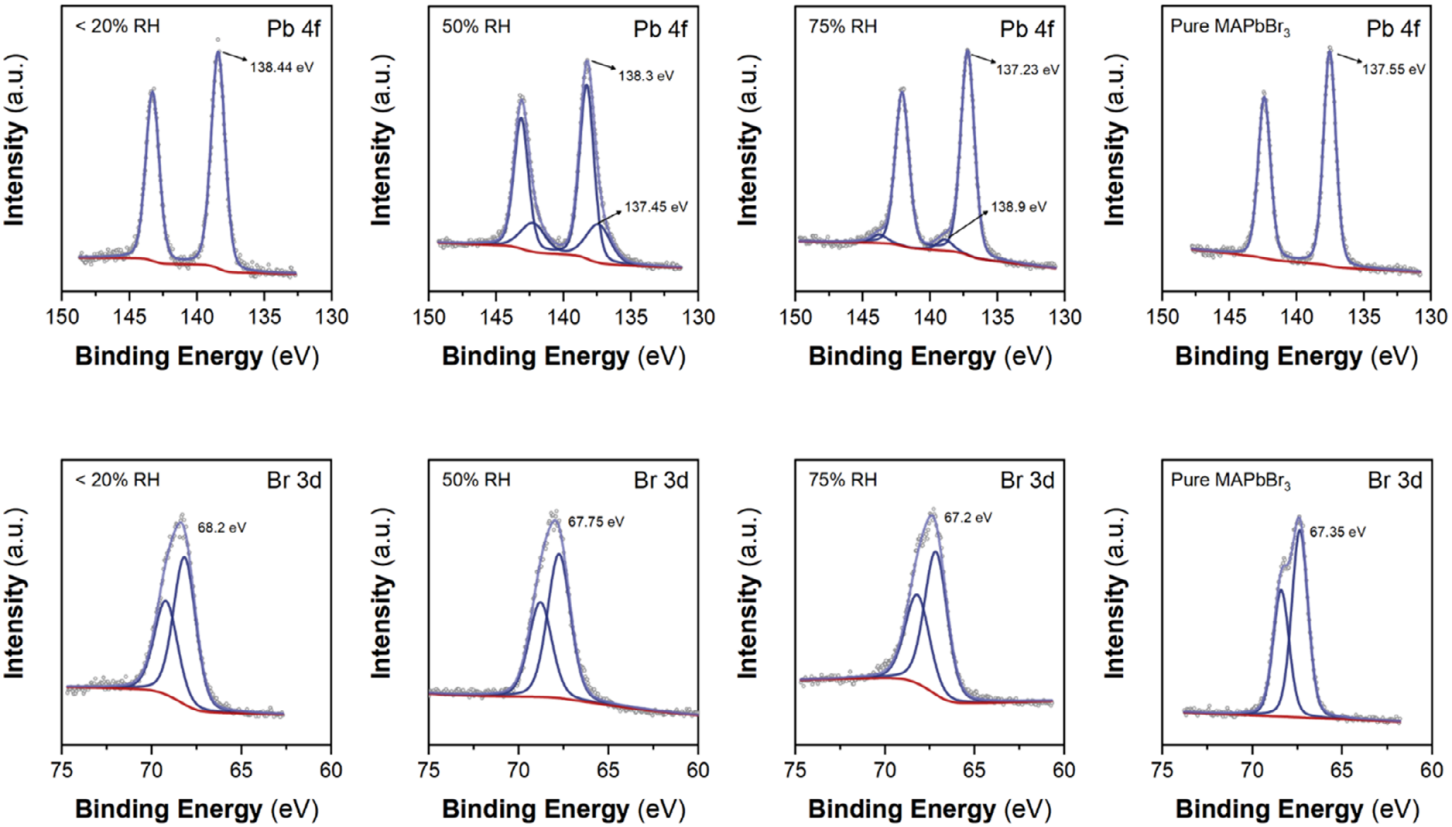
XPS Fittings for Pb 4*f* and Br 3*d* cores for different RH stages of the MAPbBr_3_ nanocomposites and the pure MAPbBr_3_.

**Figure** **5** presents the XPS spectra of the Pb 4*f* and Br 3*d* core‐levels for the <20%, 50%, 75% RH, and the pure MAPbBr_3_ samples. The spectra can be deconvoluted by assuming Voigt line shape peaks and Shirley backgrounds, considering Pb 4*f* doublets with a spin–orbit splitting of 4.87 eV and Br 3*d* doublets with a spin–orbit splitting of 1.04 eV. The sample prepared at <20 % RH presents the Pb 4*f*_7/2_ peak at a binding energy of 138.44 eV, which characterizes the Pb–Br interaction. For the 50% RH sample, the deconvolution reveals two Pb 4*f* doublets. The lower binding energy peak at 137.45 eV corresponds to Pb–O interactions,^[^ ^45^ ^]^ while the peak at 138.3 eV represents Pb–Br interactions. As mentioned earlier, the Pb–O interactions might be related to some species participating in the crystallization, such as the PbBrOH (Figure 7, Step 2). Similarly, in the 75% RH sample, the deconvolution reveals two doublets, which are attributed to Pb–Br (137.23 eV) and Pb–OH (138.9 eV) interactions.^[^ ^46^ ^]^ For the case of pure MAPbBr_3_, which was prepared at 50% RH, the peak representing the Pb—Br bond is observed at a binding energy of 137.55 eV.

Regarding the Br 3*d* core levels, the binding energy of the Br 3*d*5/2 peak is observed at 67.35 eV for the pure MAPbBr_3_, while decreases from 68.2 to 67.75 and 67.2 eV for the samples at <20, 50 and 75% RH respectively. This gradual decrease in the binding energy observed for the Pb 4*f* and Br 3*d* peaks suggests a diminishing Pb–Br interaction.^[^ ^47^, ^48^ ^]^ Specifically, the Pb–Br interaction is the weakest for the 75% RH sample and comparable to the existing one for the pure MAPbBr_3_ sample. These characteristics align well with the presence of MA_4_PbBr_6_·2H_2_O (0D) perovskite in the sample prepared <20% RH. At 50% RH, a mixture of MA_4_PbBr_6_·2H_2_O and MAPbBr_3_ is observed. Above 75%, the sample consists almost entirely of MAPbBr_3_. To confirm this, further analysis and quantification of the XPS spectra of the Pb 4*f* and Br 3*d* core levels reveal a direct reduction in the Br/Pb ratio in samples exposed to <20, 50, and 75% RH (Table S1 and Figure S3, Supporting Information). As RH increases, the Br/Pb ratio decreases. Therefore, the Pb‐OH interactions observed in the 75% RH sample, where only the 3D perovskite phase is present, suggest an OH‐capped perovskite passivation mechanism that effectively caps Br vacancies, likely contributing to the enhanced stability and improved photoluminescence (PL). The XPS results are consistent with the proposed transformation of the nanocomposites upon water exposure, aligning with the changes observed in the optical properties analysis. The O1*s* and C1*s* core‐level spectra analysis (Figure S4, Supporting Information) provides insights into the presence of hydroxyl (O–H) groups involved in the passivation mechanism (Figure 1, Steps 1 and 4). Though, the overlap with other species, such as carbonyl groups (C=O), complicates the analysis. Further details, including the O/C=O ratio and comprehensive data, can be found in the Supporting Information.

### 2.1 OH‐Driven Passivation Mechanism

**Figure 6:**
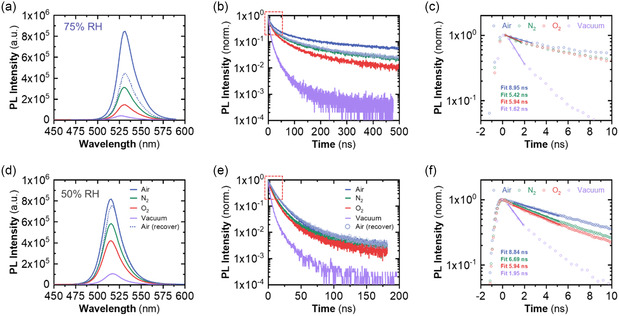
a,d) PL spectra and b,e) PL decay kinetics and c,f) insights measured for the MAPbBr_3_ nanocomposite sample at 75% (a–c) and 50% RH (d–f) in an optical flow cell with various gases at atmospheric pressure (atmospheric air at different RH, N_2_ and O_2_ from a cylinder). Vacuum measurements were carried out in a metal cryostat.

Since the PL properties of the in situ synthesized MAPbBr_3_ nanocomposites are strongly dependent on the RH during the crystallization, the key question is how different atmospheres influence the system's photophysics. To explore this dependence, we focus on the response of nanocomposite samples prepared under 75% RH (with PL maximum at 532 nm) and 50% RH (with PL maximum at 515 nm). The corresponding PL spectra and decay kinetics, sequentially measured under different atmospheres (O_2_, N_2_, air, and vacuum), are shown in **Figure** **6**. All the measurements have been performed in an optical gas flow cell see Figure S5, Supporting Information, at atmospheric pressure and with ≈30 min periods to ensure the stabilization of the emission parameters.

For both samples, the highest PL is registered under ambient/air conditions. The results obtained for nanocomposites at both RH provide strong evidence of the influence of atmospheric conditions on the PL parameters.

**Figure 7:**
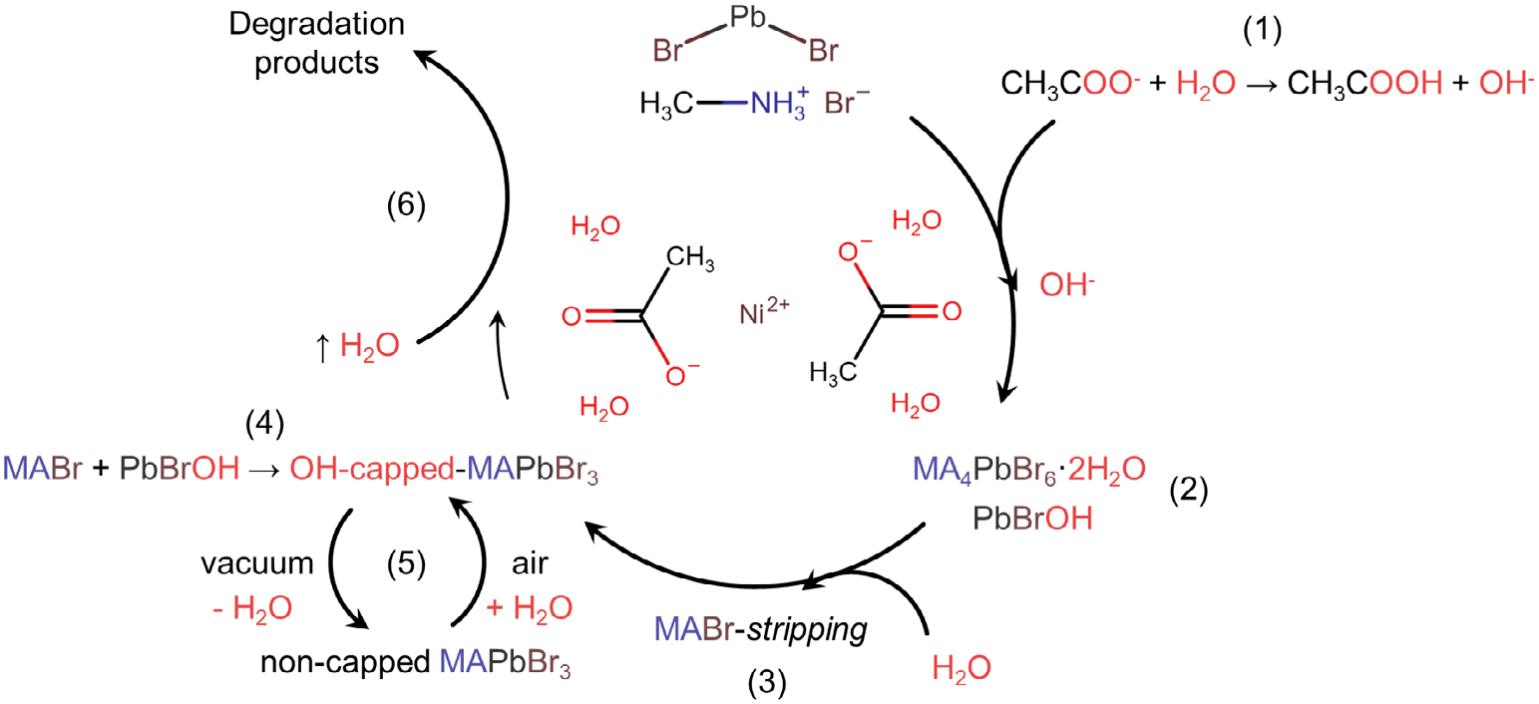
Schematic representation of the sequential chemical steps/reactions governing the crystallization mechanism of MAPbBr_3_ nanocomposites within a nickel acetate matrix. (1) Hydrolysis of acetate ions, resulting in an increased OH^−^ concentration. (2) 0D perovskite phase and PbBrOH formation. (3) MABr‐stripping mechanism induced by water interaction. (4) 0D to 3D (OH‐capped) perovskite phase transition. (5) Reversible unbinding and binding of OH‐capped perovskite NCs upon water removal. (6) Excessive water exposure leading to precursors and degradation products.

For the sample prepared at 75% RH, Figure 6a depicts how the PL intensity drops ≈3 and 6 times when the optical cell is sequentially filled with dry N_2_ and O_2_ gases, respectively. Subsequently, under vacuum conditions, the PL integral intensity decreases about 20‐fold relative to the initial intensity in air. This evolution is accompanied by an essential shortening of the PL decay kinetics (Figure 6b). When the optical cell is filled back with atmospheric air (75% RH), the PL intensity is restored to approximately half of the initial value. This partial recovery is further discussed and attributed to degradation (**Figure** **7**, Step 6), which results from prolonged exposure of the 3D NCs to a high RH of 75%.^[^ ^34^ ^]^ Moreover, we have conducted experiments to monitor the transmittance spectrum (Figure S6, Supporting Information) of the sample under both vacuum and air conditions to further assess any potential changes in absorption. No significant changes are observed throughout the cycles, indicating that the structure of the 3D NCs remains stable and does not revert to the original 0D form. Thus, all observed changes can be attributed to PL quenching.

The sample produced at a lower relative humidity (50% RH) exhibits similar PL evolution when exposed to the same cycle of atmospheres. Initially, when the atmosphere changes from air to dry N_2_ and O_2_, the PL intensity decreases by 1.4 and 1.9 times, respectively (Figure 6d). When exposed to vacuum, the integrated PL intensity drops by a factor of 6.7. These processes are also associated to shortened PL kinetics (Figure 6e). When the sample is exposed back to atmospheric air (50% RH), the PL intensity and decay kinetics nearly return to their original values (Figure 6d,e, dotted lines).

Interestingly, the reduction in PL lifetime during the transition from air to O_2_, N_2_ and vacuum (Figure 6b,e) does not show a quantitative correlation with the drop in the integral PL intensity (Figure 6a,d). The PL decay kinetics for both samples in all atmospheres, except vacuum, are highly non‐exponential, with the long‐lived components tentatively related to trapping‐detrapping processes. Therefore, we use the “primary lifetime” *τ*_pr_ as a parameter to characterize the charge recombination rate in the nanocomposites. The primary lifetime is obtained by mono‐exponential fitting of the initial decay stage. The corresponding fittings for the 75% RH sample give the “primary lifetimes” *τ*_pr_ of 8.95, 5.42, 5.94, and 1.62 ns for air, N_2_, O_2_, and vacuum conditions, respectively (see Figure 6, 7 and Table S3, Supporting Information). Interestingly, the quenching coefficients determined as *τ*_pr_(air)/*τ*_pr_(gas) from the time‐resolved measurements are 1.65, 1.51, and 5.52 for O_2_, N_2_, and vacuum, respectively. These values are essentially different (lower) than those of 2.72, 5.74, and 22.14 obtained from the integral PL rates under the same conditions. Qualitatively, analogous results are obtained for the sample at 50% RH (see Figure 6f and Table S3, Supporting Information). This suggests that the PL decay kinetics does not reflect all PL quenching processes. To explain these results, we suggest that PL quenching in the absence of the passivating effect of OH^−^ can be of two types: 1) instantaneous or static in the terminology of molecular fluorescence; and 2) dynamic. Luminescent centers subjected to instantaneous quenching do not have time to emit luminescence before being quenched. Therefore, they are visible in the luminescence decay kinetics of the entire ensemble of emitters by a drop in the amplitude of the decay kinetics. In contrast, luminescent centers subjected to dynamic quenching exhibit this quenching, depicting a reduction in the luminescence decay time but without the amplitude reduction. Simple kinetic measurements can help us determine the fraction of NCs subjected to instantaneous static quenching in the absence of passivating ligands. Indeed, if TRPL measurements in air and vacuum are performed under the same excitation and detection conditions and during the same time, then the integrals under the two kinetics should be proportional to the respective PLQY. The ratio of the integrals under kinetics measured in air and vacuum is 22.94 (Figure S7, Supporting Information), which is in excellent agreement with the ratio of the integral intensities in steady‐state PL spectra (the ratio is 22.14, see Table S3, Supporting Information). Further, the fraction of NCs that are completely quenched can be determined by measuring the reduction in the amplitude of the PL decay kinetics in vacuum compared to air, both measured under the same conditions. As can be seen from Figure S7, Supporting Information, the amplitude of the quenched PL kinetics in a vacuum is 32% of the amplitude of the PL signal in the air, which means that after pumping out adsorbed water molecules (under a vacuum), 68% of the NCs lose the PL completely by the static quenching mechanism.

In view of these results, we conclude that the removal of water from the MAPbBr_3_ nanocomposites underlines the elimination of the passivating agent responsible for the high PLQY under air/humid conditions. The extreme case is represented by the measurements in vacuum, when the greatest quenching of the PL occurs. When using gas carriers such as N_2_ and O_2_, a less pronounced decrease is observed, as these gases desorb water molecules in a lower degree and at slower rate as compared with vacuum. This trend clearly indicates that adsorbed water molecules might participate as passivating agents, in good agreement with the OH‐capped equilibrium as the passivation mechanism (Figure 7, Step 5). In contrast, these results cannot be justified in terms of the formation of the type I heterostructure (with a wider bandgap 0D phase A_4_BX_6_ or with a byproduct of the water‐induced interactions such as lead bromide hydroxide PbBrOH), a widespread explanation of the high PLQY of the 3D ABX_3_ in the literature.^[^ ^33^ ^]^ Indeed, the enhancement of PLQY due to the above‐mentioned a type I heterostructure should lead to an effective energy transfer from the 0D to the 3D phase, which is not observed in the PLE of the 3D phase: the 0D phase in the 0D/3D mixture behaves as a neutral filter absorbing radiation in the UV region, similar to the wide‐bandgap compound PbBrOH. Based on this, we conclude that the wide‐bandgap crystalline structures (0D MA_4_PbBr_6_ and PbBrOH) do not participate significantly in the increase of 3D phase PLQY by passivation. Instead, the enhancement in PLQY is primarily driven by the in‐situ generation of hydroxide ions, which effectively passivate the MAPbBr_3_ NCs.

Based on the experimental evidence and analysis, we propose the mechanism depicted in Figure 7. The mechanism begins with the formation of the 0D MA_4_PbBr_6_·2H_2_O structure, driven by the interaction between MABr and PbBr_2_ in the presence of waters of crystallization from Ni(AcO)_2_·4H_2_O (Step 2). Concurrently, PbBrOH forms as an intermediate due to OH^−^ generation by acid‐base reaction between acetates and water (Step 1). In this mechanism, acetate (CH_3_COO^−^) acts as a relatively strong base, accepting H^+^ from water and facilitating the generation of OH^−^. These OH^−^ ions then bind with the excess Pb^2+^ to form PbBrOH (Step 2) from the formation of MA_4_PbBr_6_. Attempts to use other types of nickel salts (i.e., NiSO_4_·6H_2_O, NiBr_2_·3H_2_O Ni(CH_3_SO_3_)_2_, Ni(SO_3_NH_2_)_2_·4H_2_O, Ni(PhSO_3_)_2_·6H_2_O, Ni(OH)_2_) did not yield similar results, as they lacked either waters of crystallization, the correct basicity of the counterion, or both.

As humidity increases, the “MABr‐stripping” reaction occurs, removing methylammonium bromide (MABr), which facilitates the structural transformation from 0D MA_4_PbBr_6_ to 3D MAPbBr_3_ (Step 3) by promoting the correct stoichiometry required for forming of MAPbBr_3_. Hydroxide ions (OH^−^), produced through water interactions with the acetate, then bind to the surface of the newly formed MAPbBr_3_, creating an OH‐capped 3D perovskite structure that enhances the material's stability and PL performance (Step 4). This passivation process is reversible, as the reintroduction or removal of water (vacuum), driven by the dual acid‐base character of the acetic acid/acetate system, enables the system to toggle between the non‐caped MAPbBr_3_ and the OH‐capped MAPbBr_3_ (Step 5). However, the transition from the 3D NCs back to the 0D phase is irreversible. Removing water under vacuum only quenches the PL, without affecting the sample's transmittance (Figure S6, Supporting Information). Upon extended exposure to H_2_O, the 3D MAPbBr_3_ phase ultimately degrades, resulting in the breakdown of the perovskite lattice and the perovskite degradation (Step 6). To validate this latter step, we have studied the evolution of PLQY and XRD patterns with time at 75% RH. Figure S8a, Supporting Information, shows the evolution of PLQY, while Figure S8b, displays the XRD patterns at various crystallization times. The PLQY of the nanocomposite sample reaches a maximum of ≈80% at around 30 min, followed by a progressive decline as the exposure continues. In a similar timescale, this decline is also noticeable in the XRD peak intensities as we observe a five‐fold reduction, confirming that extended exposure to high RH conditions ultimately degrades the perovskite's ionic structure.

Building on the preceding analysis, we conclude the basicity of acetate is crucial for the passivation mechanism, as it generates OH^−^ in situ, which subsequently binds to the surface of MAPbBr_3_ NCs. Its relatively strong basic character in aqueous environments allows it to effectively promote the formation of OH^−^, aiding in the stabilization and passivation of the perovskite structure. This dual acid‐base character of the acetic acid/acetate system is key to driving the hydroxide‐mediated reactions in the system.

## 3 Conclusions

In summary, we present an improved understanding of the crystallization dynamics of methyl ammonium lead bromide perovskite NCs phases through an in‐situ synthesis under controlled humid conditions. Unlike the typical H_2_O quenching effect on metal halide perovskites, we prove that humidity, particularly at levels above 20% RH, promotes the formation of 3D perovskite NCs from their former 0D structures. At lower humidity levels, the synthesis predominantly yields non‐emissive 0D perovskite and PbBrOH, while at higher RH the generation of the 3D NCs occurs. A comprehensive analysis of the PLE spectrum reveals no significant energy transfer, indicating that the 0D phase and PbBrOH function as neutral UV filters. Based on this, we conclude that the wide‐bandgap crystalline structures (0D MA_4_PbBr_6_ and PbBrOH) do not participate significantly in the increase of 3D phase PLQY by passivation. Instead, the enhancement in PLQY is primarily driven by the in situ generation of hydroxide ions, which effectively passivate the MAPbBr_3_ nanocrystals. These results underscore the critical role of water and the basicity of acetates in controlling the crystallization dynamics and generating OH^−^ that serve as NCs passivators enhancing their optical properties. Detailed PL and PL decay kinetic studies of MAPbBr_3_ nanocomposites exposed to different gas environments prove that the reversible removal of water molecules from the NCs leads to significant PL quenching, further indication that the in situ‐generated OH^−^ plays a key role in enhancing the stability and optical performance of perovskite NCs. These studies will pave the way for a deeper understanding of the role of passivating agents in deploying perovskites for applications in optoelectronic devices.

## 4 Experimental Section

4.1

4.1.1

##### MAPbBr_*3*_ Nanocomposites Preparation

To synthesize Ni(AcO)_2_–MAPbBr_3_ thin films, a single‐step method was employed based on the technique described by Noguera‐Gómez et al.^[^ ^34^ ^]^ Nickel acetate tetrahydrate (Ni(CH_3_COO)_2_·4H_2_O, 98%, Alfa Aesar) was used as the matrix precursor, while methylammonium bromide (CH_3_NH_3_Br, 99.5%, Ossila) and lead(II) bromide (PbBr_2_, 98.0%, Sigma‐Aldrich) served as the precursors for the perovskite NCs. N,N‐dimethylformamide (DMF, anhydrous, 99.8%, Sigma‐Aldrich) acted as the solvent. The standard formulation for the Ni(AcO)_2_ precursor involved dissolving 10 mmol of Ni(AcO)_2_ in 5 mL of DMF, creating a 2 m solution. This precursor solution was aged in a dry bath at 70 °C for 10 min. During this period, Ni(AcO)_2_ underwent hydrolysis and condensation (sol–gel transition), resulting in the formation of soluble colloidal clusters (branched metal‐organic polymers) and an increase in solution viscosity. The MAPbBr_3_ precursor was prepared by dissolving 5 mmol of MABr and 5 mmol of PbBr_2_ in 5 mL of DMF.

The two precursor solutions, Ni(AcO)_2_ and MAPbBr_3_ were mixed in the specific to achieve the specific molar ratios of 1 and 0.25 m respectively, and applied as ink via spin‐coating. Prior to deposition, Quartz substrates (Sigma‐Aldrich) were cleaned by ultrasonication in a 3 m HCl solution (prepared from HCl, 35 vol%, VWR Chemicals) for 10 min, followed by rinsing with a mixture of isopropanol (99.5%, VWR Chemicals) and acetone (C_3_H_6_O, 99%, VWR Chemicals) in a 3:1 ratio. The substrates were then rinsed with deionized water (Millipore) and dried with compressed air. An ozone treatment was applied to the substrate‐holding face for 10 min using a UV chamber (Ossila Ozone Cleaner) to remove organic matter.

The synthesis process involved depositing the mixed solution onto glass and spin‐coating it at 3000 rpm for 40 s in a humidity‐free environment (Drybox). During spinning, the MAPbBr_3_ nanocomposite gradually dry out. The crystallization of perovskite NCs is dependent on the concentration but for this work the precursors concentration was fixed as mentioned in the discussion part (MABr, PbBr_2_, and Ni(AcO)_2_) and we varied the relative humidity at which we exposed the samples RH.

Humidity chambers were designed (as reported previously Noguera‐Gómez et al.)^[^ ^35^ ^]^ to generate a specific humidity level ranging from 20 to 75% RH by using a supersaturated solution of different inorganic salts. The salt solutions are used because they have a well‐defined equilibrium vapor pressure at a given temperature, which makes it possible to generate a specific level of relative humidity inside a chamber. Each MAPbBr_3_ nanocomposites is hold at the desired humidity level to trigger the crystallization process for 10 min time.

##### Structural, Optical and Electronic Characterization

Field emission scanning electron microscopy (SEM) performed with an S‐4800 instrument from HITACHI (Tokyo, Japan) operating at 20 kV was used for the morphological characterization of the samples. Their crystalline structure was assessed by X‐ray diffraction (XRD) collected on a Bruker D8 Advanced X‐ray diffractometer with copper Kα radiation (*λ* = 1.5418 Å) operating at a grazing incidence of 1°, at a scan rate of 5° min^−1^ for 2*θ* angles ranging from 20° to 80°. ATR‐FTIR measurements were performed using an Agilent Cary 630 FTIR spectrometer. Transmission electron microscopy (TEM) images were obtained using a Hitachi HT7800 microscope equipped with a high‐resolution LaB6 filament, operating at an acceleration voltage of 100 kV. TEM samples were prepared by dispersing crushed MAPbBr_3_ nanocomposites on top a Carbon coated (Cu/C) TEM grid using methyl acetate as a solvent. Spectrophotometer UV‐Vis V‐760 from Jasco was used for the UV‐Vis measurements. Steady‐state PL and PL excitation, as well as time‐resolved PL (TRPL) measurements of the samples were performed with the use of Edinburgh Instrument FLS 1000 fluorimeter with excitation and detection double grating Czerny‐Turner monochromators, the detection one directed the spectrally selected PL light into high‐speed PMT in a cooled housing with the instrument response of about 200 ps coupled with a photon‐counting system. A 150W ozone‐free xenon arc lamp was used as a light source for steady‐state measurements and a pulsed 405 nm solid state laser with pulse duration of about 1 ns for TRPL measurements. X‐ray photoemission spectroscopy (XPS) Characterization: High‐resolution XPS measurements were performed in a SPECS GmbH system (base pressure 1.0 × 10^−10^ mbar) equipped with an ASTRAIOS 190 2D‐CMOS hemispherical analyser. Photoelectrons were excited with the Al–K_α_ line (1486.7 eV) of a monochromatic X‐ray source μ‐FOCUS 500 (SPECS GmbH). Measurements were taken at room temperature with a pass energy of 50 eV.

## Conflict of Interest

The authors declare no conflict of interest.

## Author Contributions

**Jaume Noguera‐Gómez**: data curation (lead); investigation (lead); methodology (equal); writing—original draft (lead). **Víctor Sagra‐Rodríguez**: data curation (supporting); investigation (equal); methodology (supporting). **Vladimir S. Chirvony**: formal analysis (lead); investigation (lead); writing—original draft (equal); writing—review & editing (equal). **Miriam Minguez‐Avellan**: data curation (equal); investigation (equal). **Mahesh Eledath Changarath**: data curation (supporting); investigation (supporting). **Juan F. Sánchez‐Royo**: formal analysis (supporting); writing—review & editing (supporting). **Juan P. Martínez‐Pastor**: resources (supporting); writing—review & editing (supporting). **Pablo P. Boix**: conceptualization (lead); formal analysis (lead); funding acquisition (lead); methodology (lead); resources (lead); supervision (lead); writing—review & editing (lead). **Rafael Abargues**: formal analysis (lead); funding acquisition (lead); methodology (supporting); resources (equal); supervision (equal); validation (lead); writing—review & editing (equal).

## Supporting information

Supplementary Material

## Data Availability

The data that support the findings of this study are available from the corresponding author upon reasonable request.
