# Supplementary material for: Passivation Mechanism in Highly Luminescent Nanocomposite‐Based CH3NH3PbBr3 Perovskite Nanocrystals
Source: Small Sci. 2025 Jan 22;5(5):2400529. doi: 10.1002/smsc.202400529 (PMC12087776; doi:10.1002/smsc.202400529)
Supplement: Supplementary file 1 — Supplementary Material [file SMSC-5-2400529-s001.pdf]

Supplementary information

Passivation Mechanism in Highly Luminescent Nanocomposite-based $CH_3NH_3PbBr_3$ Perovskite Nanocrystals

Jaume Noguera-Gómez, Víctor Sagra-Rodríguez, Vladimir S. Chirvony*, Miriam Minguez-Avellan, Mahesh Eledath Changarath, Juan F. Sánchez-Royo, Juan P. Martínez-Pastor, Pablo P. Boix,* Rafael Abargues*

Morphological Characterization

Regarding the layers morphology, the scanning electron microscopy (SEM) images reveal that the $Ni(AcO)_2$-$MAPbBr_3$ nanocomposites (Figure S1c) present a uniform and dense structure. Cross-sectional studies show ~300 nm (Figure S1a) while in top-view captures (Figure S1b) the $MAPbBr_3$ nanocomposites reveal highly smooth surfaces.

Prior their deposition in copper TEM Grids, these native structures are compromised during the standard sample preparation process, which involves scraping the material from the substrate, dispersing it in an organic solvent, and subsequently applying ultrasonic treatment. Repeated efforts to obtain transmission electron microscopy (TEM) images of as-prepared $MAPbBr_3$ nanocomposites structures (< 20 % RH) were unsuccessful. Figure 4a shows TEM images of $MAPbBr_3$ nanocomposites upon exposure to 75% RH. The images depicted black-doted shaped nanoparticles of different sizes, which we associate with 3D $MAPbBr_3$ perovskites. TEM images do not reveal the presence of any additional structures unlike what confirmed hereafter by Absorption and XPS analyses. Factors such as samples' manipulation or the beam electron destructive effect might hinder the observation of native multicomponent structures. This issue is discussed in more detail hereforth.

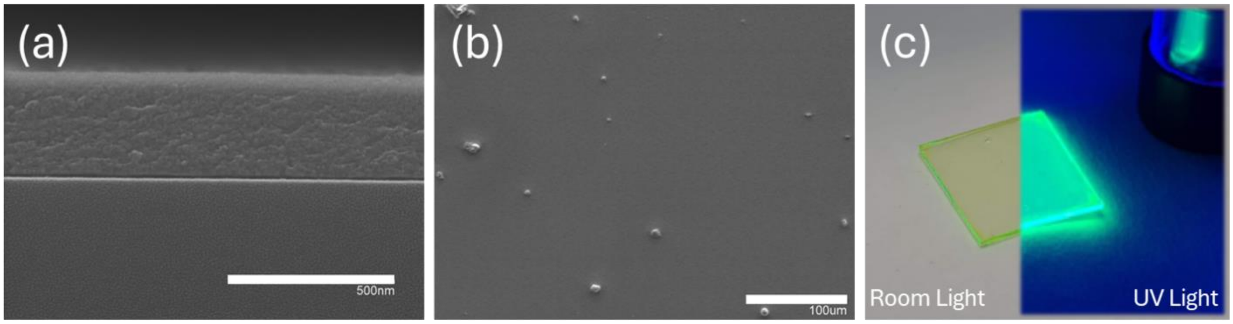

Figure S1. (a) Cross-sectional at 30º angle perspective (b) Top-view SEM Images and (c) photograph under different illumination of the $MAPbBr_3$-$Ni(AcO)_2$ nanocomposites.

Structural Characterization

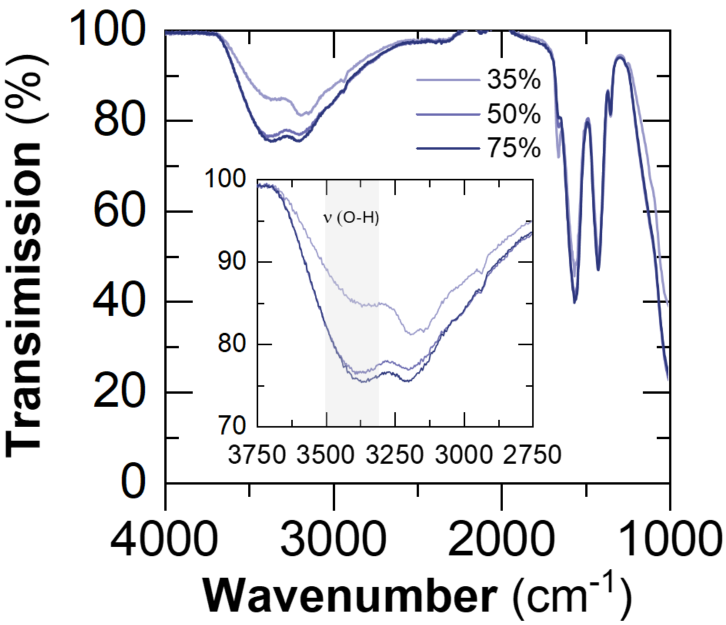

Figure S2. ATR-FTIR spectra comparing $MAPbBr_3$ nanocomposites at the different RH studied conditions.

Table S1. Stoichiometry parameters based on XPS Fit analyses for the Pb 4f and Br 3d cores. Br/Pb stoichiometric ratio calculations.

Sample (RH)	Peak Area		Atomic percentage		Stoichiometry
	Pb 4f	*Br 3d*	*Pb 4f*	*Br 3d*	

< 20 %	1231.57	862.08	15.14	84.86	Pb $Br_{5.6}$
50%	2489.71	1349.07	18.73	81.27	Pb $Br_{4.33}$
75%	2781.49	1333.39	20.67	79.33	Pb $Br_{3.83}$
Pure $MAPbBr_3$	2132.08	937.73	22.12	77.88	Pb $Br_{3.52}$

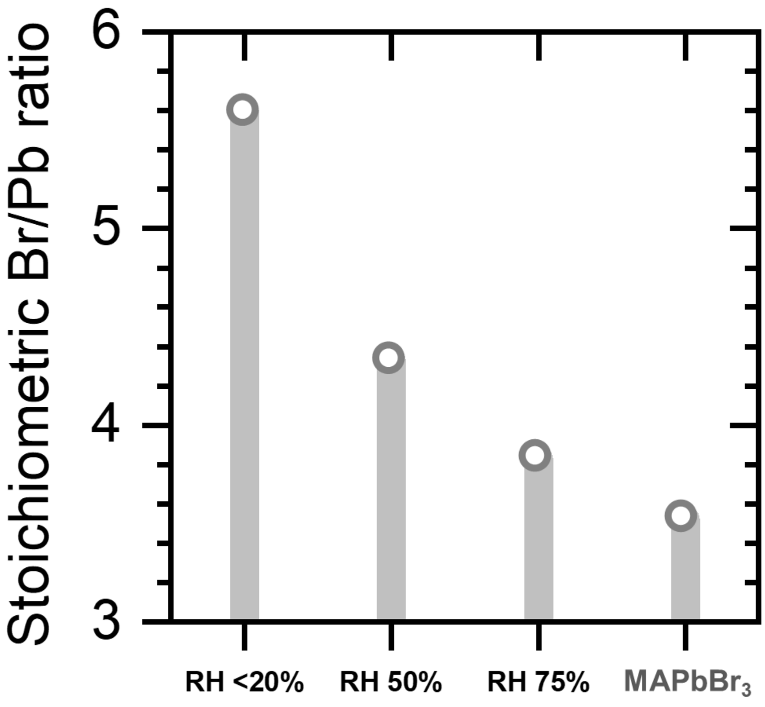

Figure S3. Stoichiometric ratio among Br/Pb calculated from the XPS data.

The study of O1s and C 1s core-level spectra (Figure S3) can give some light to elucidate the presence of hydroxyl (O-H) groups involved in the passivation mechanism (Figure 1, Steps 1 and 4). As it can be seen in Figure S3, the O 1s core-level peak in the pure $MAPbBr_3$ perovskite is dominated by a single component at 532.3 eV attributable to OH groups that exist upon preparation in humid atmospheres.[49] On the other side, the C 1s core-level spectra acquired in this sample reveals the presence a single peak at 285.0 eV attributable to the presence of adventitious Carbon

in the sample surface. For NCs grown in acetate-rich environments, O 1s XPS spectra reveals the presence of an intense and relatively wide peak located, as in the one observed in pure perovskites, at energies ~532 eV. Only a small additional O 1s signal has been resolved in samples prepared under 50% RH, attributable to Pb-O local environments. The observation of the O 1s peak at 532 eV in the XPS spectra acquired in the samples grown in acetate-rich environments may be tentative considered as evidence of the incorporation of O-H groups during sample preparation. However, it cannot be discarded the presence of other oxidized species in the sample surface. In fact, the C 1s spectra measured in these samples reveal the presence of a prominent component attributed to carbonyl carbon (C=O) from the acetate precursors. An eventual O 1s XPS signal coming from C=O groups would overlap with that from O-H groups. In order to discriminate the presence of O-H groups in samples prepared in acetate-rich environments, we have calculated the atomic $O/C_{C=O}$ ratio, as obtained by XPS by considering the XPS integrated intensity of both the C 1s peak attributed to C=O ($C_{C=O}$) and the main O 1s peak. Results obtained (Table S2) clearly reveal that a large fraction of the main O 1s XPS peak observed in in samples prepared in acetate-rich environments stem from O-H, which would support the incorporation of hydroxyl (O-H) groups involved in the passivation mechanism.

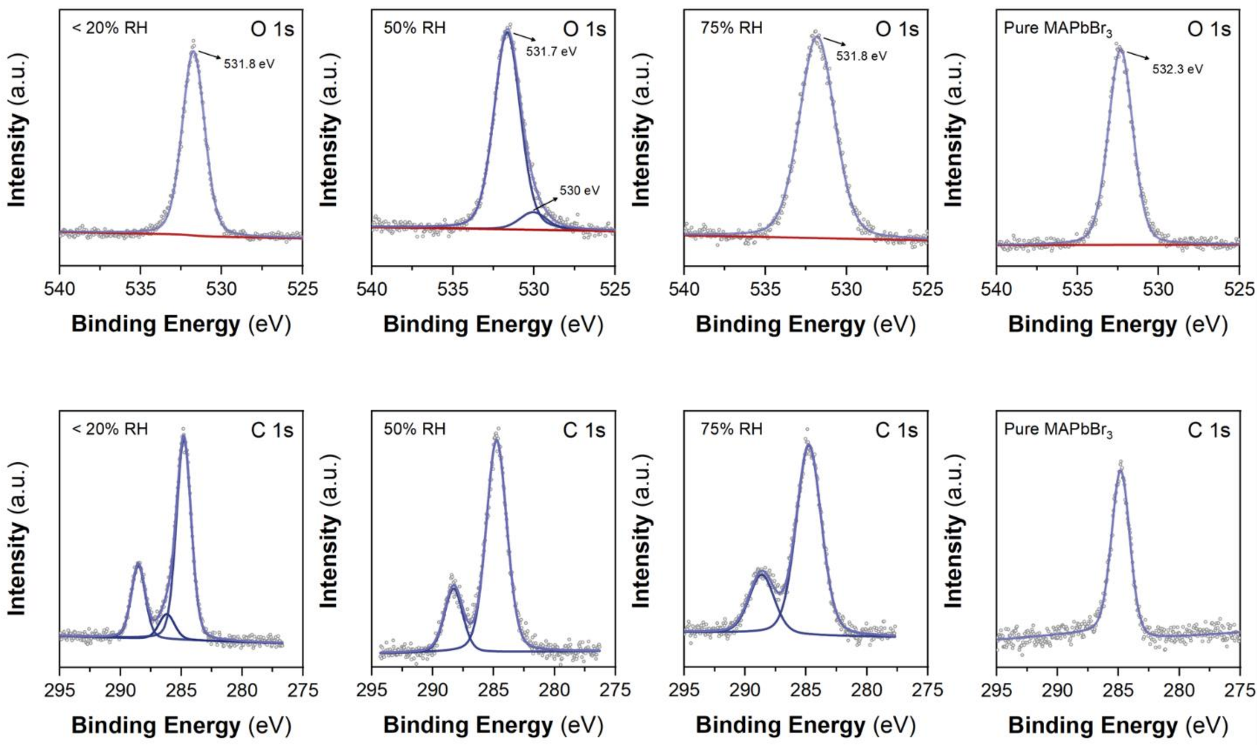

Figure S4. O 1s and C 1s core-level spectra acquired by XPS in $MAPbBr_3$ nanocomposites and pure $MAPbBr_3$ at different RH.

Table S2. Stoichiometry parameters based on XPS Fit analyses for the C 1s O 1s core-level lines cores attributable to acetate groups.

Sample (RH)	Peak Area		Atomic percentage		O/C Atomic ratio
	C 1s (C=O)	*O 1s*	*C 1s (C=O)*	*O 1s*	
< 20 %	244.47	3374.45	17.5	82.5	4.71
50%	420.41	2165.1	36	64	1.77
75%	534.6	2975.6	34	66	1.94

On the passivation mechanism

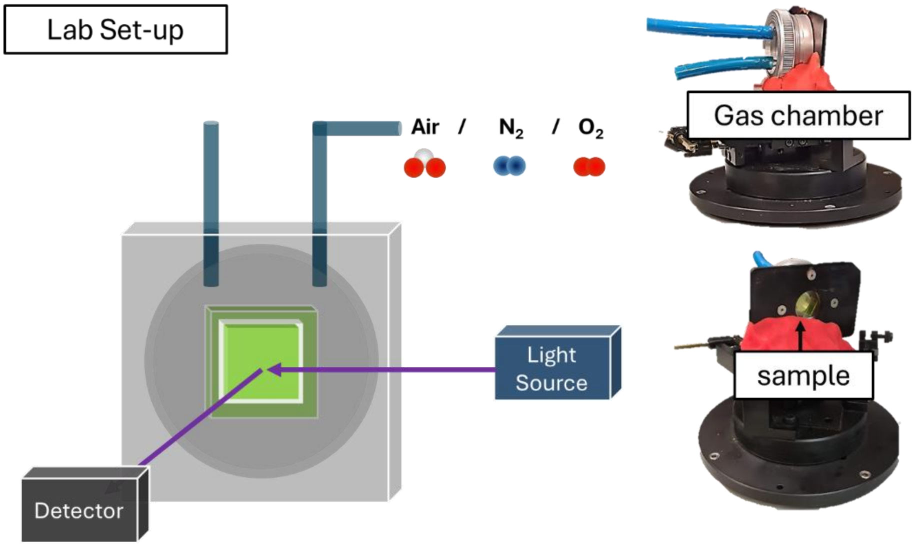

Figure S5. Lab Gases Set-up for passivation dynamics studies.

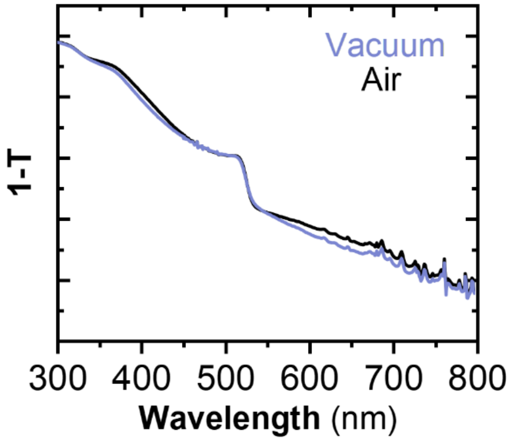

Figure S6. 1-Transmittance of a $MAPbBr_3$ nanocomposite at 75%RH under vacuum and at air conditions.

Table S3. PL Decay monoexponential fittings and PL Integral Intensity of $MAPbBr_3$ nanocomposite samples form Figure 6.

	Air	*N2*	*O2*	*Vacuum*
75% RH τ_{pr} (ns)	8.95	5.42	5.94	1.62

75% RH τ_{pr} (air)/ τ_{pr}	1	1.65	1.51	5.52
75% RH PLint(air)/Plint	1	2.72	5.74	22.14
50% RH τ_{pr} (ns)	8.84	6.69	5.94	1.95
50% RH τ_{pr} (aekir)/ τ_{pr}	1	1.32	1.49	4.53
50% RH PLint(air)/Plint	1	1.4	1.9	6.7

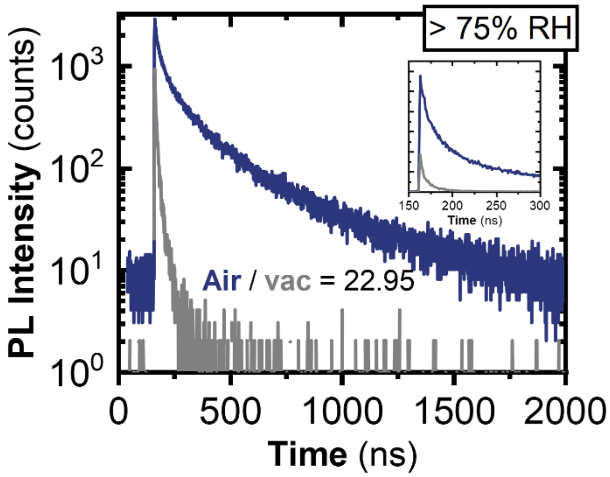

Figure S7. PL Decay kinetics insight for a sample at 75%RH operating at the same excitation and detection conditions.

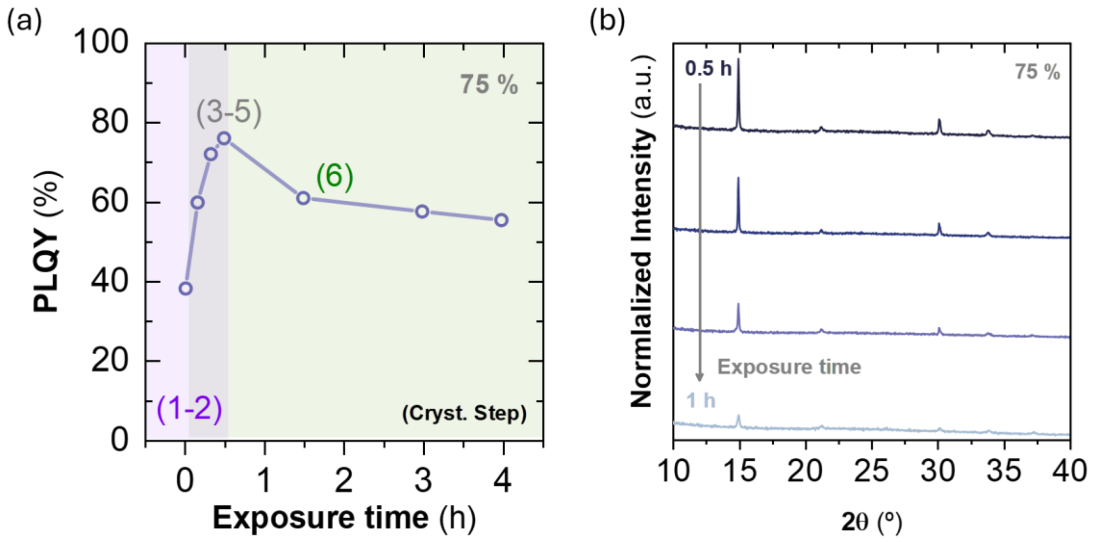

Figure S8. Crystallization dynamics monitoring the evolution of PLQY and XRD profiles of a $MAPbBr_3$ nanocomposite at 75 % RH.
